# Obstetrics and Gynæcology

**Published:** 1893-06

**Authors:** 


### OBSTETRICS AND GYNECOLOGY.

EDITED BY DR. VIRGIL O. HARDON.

### ARTIFICIAL INDUCTION OF LABOR.

By H. Fehling (*Berl. Klin. Wochen.*, 1892).

The author hopes by the improved methods of antisepsis or asepsis to reduce the number of Cæsarean sections by artificial induction of labor. The time for the induction of labor should not be before the thirty-fifth and not after the thirty-seventh week. A contracted pelvis is the most frequent condition which calls for the induction of labor, particularly flat pelvis.

The operation is not so satisfactory in a generally contracted pelvis. A careful examination is in all cases necessary. Particular attention should be given to the fact that in multiparæ there is usually an increase in the weight of each successive fetus. The operation should not be practiced where a pelvis is less than seven cm. in its true conjugate.

In light cases of osteomalacia normal delivery may be expected, since the pelvis may admit of some stretching. Further indications for the induction of artificial labor may be recognized in cases of retrocervical fibroids, and all tumors of the bony pelvis, as also in cases of marked inflammatory masses situated in the pelvis; the cases, however, which call chiefly for this treatment are those of chronic parenchymatous interstitial nephritis.

Cardiac diseases rarely produce symptoms sufficient to consider this operation; on the other hand, however, advanced cases of tuberculosis may serve as an indication.

A careful measurement of the pelvis is in all cases the most important, and the theories of Muller, who states that the induction of labor should not be practiced as long as the child's head can pass into the pelvic cavity, deserve careful attention, as well as that of Prochownick, who places stress upon an anti-fat treatment, which consists in avoiding all carbo-hydrates and fluids.

In order to facilitate the dilatation of the os internum he advises warm, not hot, vaginal douches, six to twelve litres, twice daily.

If this remains without the desired result an elastie bougie should be passed into the uterus and allowed to remain there several days, in case no fever presents itself.

If the pains have set in, a normal spontaneous delivery may be expected, and all operative procedures are contraindicated unless fever should set in.—*Med. & Surg. Reporter.*

---

### THE SIGNIFICANCE OF VAGINAL DISCHARGES.

A leucorrhœa inodorous or of mild odor, persisting during the climacteric accompanied by increasing hemorrhage, is suspicious, and demands investigation.

A leucorrhœa profuse, of peculiarly fetid odor, grumous, excoriating, appearing early or late during the climacteric, with profuse hemorrhage, is reasonable evidence of cancer of the cervix.

A leucorrhœa, moderate in amount, ill-smelling (the peculiarly fetid odor of cancer of the cervix being absent), accompanied by hemorrhage, suggests cancer of the corpus uteri.

A leucorrhœal discharge, with hemorrhage containing material like the washings of meat, is said to indicate sarcoma.

A watery discharge, as a rule, occurring during menstruation, odorless or of little odor, persisting, accompanied by profuse hemorrhage, indicates fibroids; with little or no hemorrhage, polypi. Profuse bloody discharges coming on gradually with declining menstruation, ceasing usually with the menstrual flow, point to fibroids. Persistent profuse discharges of blood occurring spontaneously, arising from sudden exercise or coition, occurring, as a rule, after the menopause, indicate cancer.

A gradually increasing amount of menstrual flow is suspicious and needs investigation. "Post-climacteric hemorrhages in a fibroma of the uterus of long standing form one of the principal grounds for the suspicion of sarcoma." (Borner.)

The early recognition of malignant disease and the possible prevention of that fatal exhaustion which accompanies it by the administration of drugs, and the application of those methods which, in a measure, may be supposed to offset the terrific drain on the nervous system, inasmuch as present experience shows that early removal of diseased tissue prolongs life, and the importance of early diagnosis and treatment can hardly be overestimated.—*N. E. Med. Gazette.*

---

DR. H. S. HOUGHTON says, in a recent number of the New York *Medical Journal,* that one must look upon the patient who has just passed through the third stage of labor as a sufferer of traumatism, has been wounded, and, therefore, her case belongs to the domains of surgery. As the first law of surgery is rest, so the lying-in period is a period of rest, so modified that the patient emerges from it a well and perfectly healthy woman. After the birth of the child, the mother must look forward to a period of six weeks of convalescence devoted solely not only to getting well, but to getting sound and strong. This period is divided into two weeks of absolute rest in bed, one week of alternate resting in bed and moving about the room; the fourth, in gaining the effects of fresh air and sunlight, and the last two in gradually resuming the household duties. By carrying out this plan, as given in detail in the paper, the writer says we may hope to do away with the too frequent evil consequences of childbirth.—*Med. and Surg. Rep.*

